# Assessment of the Carotid Bodies in Magnetic Resonance—A Head-to-Head Comparison with Computed Tomography

**DOI:** 10.3390/diagnostics13050993

**Published:** 2023-03-05

**Authors:** Lukasz Budynko, Tomasz K. Nowicki, Mariusz F. Kaszubowski, Dominik Swieton, Maciej Piskunowicz

**Affiliations:** 1 Department of Radiology, Faculty of Medicine, Medical University of Gdansk, Smoluchowskiego 17, 80-214 Gdansk, Poland; 2 2nd Department of Radiology, Faculty of Health Sciences with the Institute of Maritime and Tropical Medicine, Medical University of Gdansk, Smoluchowskiego 17, 80-214 Gdansk, Poland; 3 Department of Statistics and Econometrics, Faculty of Management and Economics, Gdansk University of Technology, Traugutta 79, 80-233 Gdansk, Poland

**Keywords:** carotid body, magnetic resonance imaging, computed tomography, Dixon sequence

## Abstract

Objectives: To evaluate carotid body visibility in contrast-enhanced magnetic resonance (MR) studies and to compare the results to contrast-enhanced computed tomography (CT). Methods: Two observers separately evaluated MR and CT examinations of 58 patients. MR scans were acquired with contrast-enhanced isometric T1-weighted water-only Dixon sequence. CT examinations were performed 90 s after contrast agent administration. Carotid bodies’ dimensions were noted and their volumes calculated. To quantify the agreement between both methods, Bland–Altman plots were computed. Receiver operating characteristic (ROC) and its localization-oriented variant (LROC) curves were plotted. Results: Of the 116 expected carotid bodies, 105 were found on CT and 103 on MR at least by a single observer. Significantly more findings were concordant in CT (92.2%) than in MR (83.6%). The mean carotid body volume was smaller in CT (19.4 mm^3^) than in MR (20.8 mm^3^). The inter-observer agreement on volumes was moderately good (ICC (2,k) 0.42, *p* < 0.001), but with significant systematic error. The diagnostic performance of the MR method added up to 88.4% of the ROC’s area under the curve and 78.0% in the LROC algorithm. Conclusions: Carotid bodies can be visualized on contrast-enhanced MR with good accuracy and inter-observer agreement. Carotid bodies assessed on MR had similar morphology as described in anatomical studies.

## 1. Introduction

The carotid body (CB) lies in the common carotid artery adventitia, measuring 5–7 mm in height and 2.5–4 mm in width [1]. CBs constitute a cluster of specialized non-neuronal chemoreceptor tissue [2], detecting changes in arterial blood gases concentration and stimulating the respiratory centre in the brain stem [3]. Currently, there is considerable interest in the adverse effects of chronic overactivity of CBs, which has been linked to several sympathetically mediated diseases: chronic heart failure (CHF), arterial hypertension (HT), and diabetes mellitus (DM) [4]. Autopsy studies indicated CB volume increases in the settings of CHF and HT, an effect related to cell hyperplasia driven by an oxygen-depleted state [5]. Early attempts to reduce CB activity in respiratory diseases by surgical excision did not bring the desired results. However, a reduction in blood pressure was observed, which stimulated further efforts to decrease their sympathetic activation in CHF and HT [6].

At present, CB visualization relies on computed tomography angiography (CTA) [7,8], exposing a patient to high radiation doses and the possible adverse effects of intravenous iodine-based contrast agent (CA) administration. There are also two reports in the literature concerning the detection of CBs by ultrasound [9,10]. Meanwhile, magnetic resonance (MR) has become an essential imaging modality in the head and neck region due to its excellent tissue contrast and comprehensive tissue characterization. Considering the enhanced signal-to-noise ratio and resolution capabilities of modern high-field scanners, it should be possible to distinguish unaltered CBs in head and neck examinations.

This retrospective cohort study was aimed to evaluate CB visibility in MR head and neck studies and compare results to contrast-enhanced CT of the same patients.

## 2. Materials and Methods

### 2.1. Patients

We browsed our hospital information system database for contrast-enhanced head and neck MR and CT examinations with the following inclusion criteria: (1) patients at least 18 years of age, (2) the time period between MR and CT studies not longer than 5 years, (3) MR study protocol containing contrast-enhanced isometric sequence in T1-weighted images with Dixon technique and diffusion-weighted imaging (DWI), (4) CT slice thickness equal to or less than 1.5 mm, (5) arterial or arteriovenous phase included in CT examination, (6) coverage of the carotid bifurcation in both examinations. We excluded studies with considerable motion artefacts in the region of interest and with neck masses infiltrating carotid arteries. Necessary images were downloaded from the hospital PACS server.

### 2.2. Magnetic Resonance Examination Protocol

MR images were acquired on a 1.5 T scanner (Magnetom Aera, Siemens, Erlangen, Germany). We assessed the CBs in the contrast-enhanced 3D acquired gradient-echo, volumetric interpolated breath-hold examination (VIBE) sequence in T1-weighted images with the Dixon technique. Echo time (TE) was 2.4 ms, and repetition time (TR) 7.6 ms. Images were acquired with a parallel imaging technique (GRAPPA, acceleration factor of two) and the number of averages of two. The acquisition voxel size was 1.18 × 1.0 × 1.27 mm, and the reconstructed voxel was isometric and measured 1.0 × 1.0 × 1.0 mm.

An automatic pressure injector was used for the administration of CA. The total volume of administrated CA was 0.5 or 1.0 mL/kg of the patient’s body weight, depending on the manufacturer’s recommendation. CA administration with a flow rate of 3 mL/s was followed by a flush of 30 mL of normal saline at the same rate.

**Figure 1:**
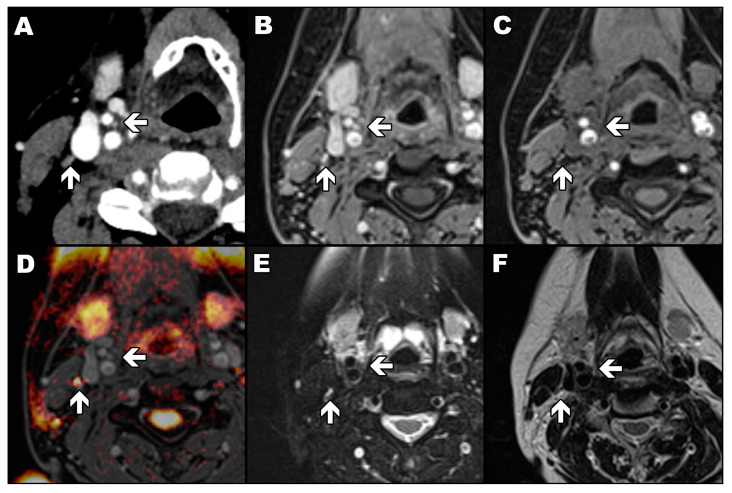
Signal characteristics of the carotid body and lymphatic node. Arrows mark the carotid body (←) and lymph node (↑). Axial contrast-enhanced computed tomography image of the patient with marked carotid body and lymph node (**A**); VIBE sequence in T1-weighted image with Dixon technique in the water-only image with (**B**) and without (**C**) contrast agent enhancement; diffusion-weighted image with *b* value of 1000 s/mm^2^ automatically fused with contrasted-enhanced VIBE sequence in T1-weighted image with Dixon technique in the water-only image (**D**); turbo spin-echo sequence in T2-weighted image with (**E**) and without (**F**) fat saturation.

The readout-segmented echo-planar diffusion-weighted sequence with *b* values of 0, 500 and 1000 s/mm^2^, slice thickness of 5.0 mm, gap of 1.0 mm, and pixel size of 1.5 × 1.5 mm was acquired with parallel imaging technique (GRAPPA, acceleration factor of two). The sequence was used only to differentiate CBs from potentially adjacent lymph nodes (Figure 1). Only a diffusion-weighted image allows a lymph node to be easily distinguished from a carotid body as the first one has a high signal (yellow) and the second has a low signal (Figure 1D). In the rest of the presented magnetic resonance images, a lymph node and a carotid body have a similar appearance.

### 2.3. Computed Tomography Protocol

Protocols for contrast-enhanced head and neck CT varied moderately between studies. Images were obtained on either a 128-row CT scanner (Somatom Definition Flash, Siemens) or a 64-row CT scanner (Lightspeed VCT XT, GE Healthcare, Chicago, IL, USA) available at our institution. Patients received 80 mL of CA, and the iodine concentration was either 350 or 400 mg/mL. The bolus of CA (1.0 mL/s) was followed by a flush of 30 mL of normal saline (3.0 mL/s). The acquisition started 90 s after the initiation of the CA bolus. Images were acquired in the arteriovenous phase. The helical acquisition of 0.6 or 0.625 mm collimated images with a pitch of 0.53–0.8 was used. The kilovoltage was within a range of 100 to 140 kV with a standard reference output of 110 mAs up to 280 mAs. The automated dose reduction programs were performed on both scanners. The reconstruction field of view ranged from 240 to 280 mm, and the reconstruction matrix was 512 × 512, resulting in pixel size from 0.47 to 0.55 mm. Reconstruction slice thickness was from 0.6 to 1.5 mm, depending on the reconstruction algorithm. Images were reconstructed with soft tissue algorithms provided by manufacturers.

### 2.4. Carotid Bodies Identification

Anonymized studies were evaluated independently with a one-week interval between MR and CT reading sessions. Two researchers (a fourth-year radiology resident and a radiology specialist with five years of expertise in head and neck imaging) read scans separately on dedicated workstation software (Syngo.via VB20A, Siemens). MR studies were viewed in a standard window. Consistent window level settings (width 190, centre 90) were used in the case of CT images.

**Figure 2:**
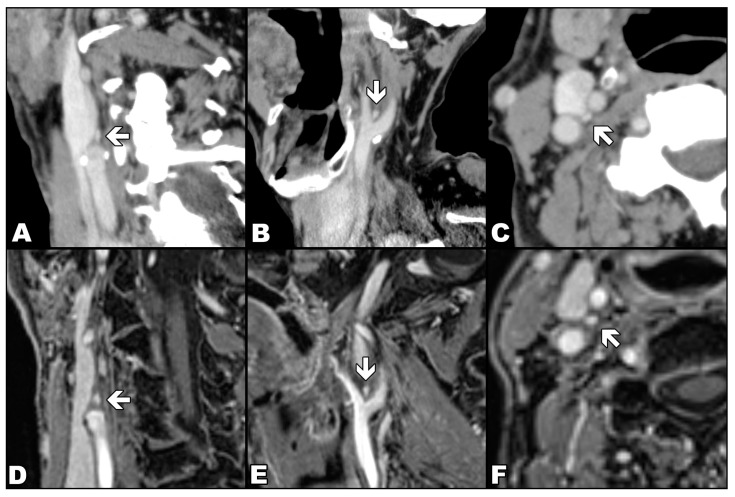
Comparison of the appearance of the carotid body (arrows) in contrast-enhanced computed tomography (upper row) with the appearance in contrasted-enhanced VIBE sequence in T1-weighted image with Dixon technique in the water-only images (lower row). Computed tomography (images (**A**–**C**)) and magnetic resonance (images (**D**–**E**)) examinations show the carotid body in three orthogonal planes: oblique coronal (**A**,**D**), oblique sagittal (**B**,**E**) and axial (**C**,**F**).

As in previous studies [7,9], CB was defined as a reproducible, ovoid, avidly enhancing structure at the inferomedial aspect of the carotid bifurcation (Figure 2). In the case of MR readings, each researcher used a semi-quantitative confidence scale from 1 to 6 points, representing the number of typical features displayed by the assessed CB and, therefore, the probability that the detected focus represents a CB. The typical features were: location adjacent to the carotid bifurcation, clearly separated oval or flame-shaped structures, transverse axis from 2 to 4 mm and longitudinal axis up to 8 mm, marked enhancement after CA, but lesser than arterial lumen. One point was assigned if no CB was visible, and six points if a single structure with all typical features of CB was detected (Table 1). A detailed assessment protocol is available in Supplementary Materials File S1.

After annotating CBs on all datasets, researchers first compared their CT findings to establish a reliable reference. Each difference deemed significant (CB location mismatch, size discrepancy more than 50%) was thoroughly discussed. After correcting obvious mistakes, a consensus was reached on the most probable CB location. MR findings were later evaluated against our mutual CT agreement. If a finding in MR was not visible in CT (the reference method), then it was not considered a CB. We reported the findings in the form of binary variables under the following conditions: (1) if a particular CB was visible in either diagnostic modality, (2) if both readers marked the CB in the same location, (3) if there was a consensus about CB localization between both methods.

### 2.5. Carotid Bodies Measurements

Orthogonal measurements of CBs were performed bilaterally on multiplanar reconstructed MR and CT images, thereby designating their locations. For the measurements in MR, solely isometric contrast-enhanced VIBE sequence in T1-weighted, water-only images was used. CB volume was calculated based on the standard formula for ellipsoids (1):

(1) $$V=\frac{1}{6}\pi \cdot x\cdot y\cdot z$$

where *x*, *y*, and *z* are transverse, sagittal, and longitudinal dimensions, respectively.

Additionally, on CT images, region of interest markers were inserted in common carotid arteries 2 cm below bifurcation to calculate CT enhancement differences.

### 2.6. Statistical Analysis

The results were analyzed with dedicated statistical software (STATISTICA 13, StatSoft, and PQStat 1.6.4.120, PQStat Software). ROC and LROC curves were computed with free web-based tools [11,12]. All calculated *p* values (with a priori significance level α set to 0.05) were two-tailed for random variables with a symmetric distribution. Normality was verified by the Shapiro–Wilk W test. Arithmetic means with 95% confidence intervals and standard deviations were calculated for quantitative variables. The reproducibility of quantitative measurements was assessed with the intraclass correlation coefficient (ICC) for concordant CBs. To quantify the agreement between both methods and evaluate distribution error, Bland–Altman plots [13] were constructed with ±1.96 SD agreement limits. To examine the significance of measurement differences between methods, Student’s *t*-test and Cochrane Q test were used, depending on variable properties.

### 2.7. Receiver Operating Characteristic

To delineate the MR ability to detect CB within set discrimination thresholds, we plotted two receiver operating characteristic (ROC) curves: conventional—as first described by Egan [14], and localization ROC (LROC)—its extension developed among others by Swensson [15]. LROC is optimized for detection tasks for multiple readers on the premise that there is only one lesion per assessed area. To fulfil the algorithm requirements, we treated each side of the neck as a separate location. Having ascertained that there was no statistical significance between the diagnostic efficiency of both readers, we collated our readings. Because CT has been assigned as the method of reference, CT studies with non-discernible CBs were removed. All the MR readings with CT references were included in the analysis.

The study was approved by the Independent Bioethics Committee for Scientific Research at Medical University of Gdansk, Gdansk, Poland (NKBBN/183/2018).

## 3. Results

We found 58 patients with adequate head and neck MR studies and recent CT scans. The mean period between acquisitions of the CT and MR scans was 481 days (median 187 days). The group consisted of 32 men and 26 women (mean age: 58 years, median 59 years, range: 21–88 years).

### 3.1. Carotid Bodies Recognition

Of the expected 116 CBs, 105 were found on CT scans at least by one reader. In one instance, an anatomical variant of the carotid bifurcation precluded identification, one CB was covered by a nearby pathologic lesion, and nine were evaluated as truly imperceptible. Associated CT sensitivity, considering all reported findings as true positives, was 90.52% (95% CI 83.67–95.17%).

In MR studies, after validation with CT results, 103 CBs were identified, yielding a sensitivity of 88.79% (95% CI 81.60–93.90%). There was no significant difference between the sensitivity of both modalities. No significant difference was observed in the visibility of CBs between both methods (Cochrane Q test, *p* = 0.64).

Concordance between observers was achieved in 107 CT readings (92.2% of the total): 97 visible instances and 10 imperceptible instances. Seven discordant CBs were not initially recognized by one of the observers, and, in two cases, each attributed CB to other structures. In comparison, there was agreement in 97 cases in MR (83.6% of the total), with 13 CBs imperceptible for one observer and six structures mismatched. There was a higher rate (Cochrane Q, *p* = 0.04) of concordant descriptions in computed tomography (agreement in 92% of cases) than in magnetic resonance (84%).

### 3.2. Carotid Bodies Dimensions

The average CB dimensions acquired from CT readings were consistently smaller in comparison with MR (Table 2). Average CB volume was estimated respectively 19.4 mm^3^ and 20.8 mm^3^ with a moderate degree of correlation between both modalities (ICC (2,k) 0.46, *p* < 0.003). Systematic bias for CB volume measurement, estimated on the base of Bland–Altman analysis, added up to 1.4 mm^3^. Although CB volumes in MR were typically reported as larger, the measurement variation seems constant and, for larger structures, contributed less to the overall result. Volume differences followed the normal distribution. However, established agreement limits were broad (20.4 mm^3^ for ±1.96 SD). There was a very good correlation between CBs volumes on both sides of the neck in both techniques (Spearman’s ranks, *p* < 0.001). Inter-observer agreement on CBs volumes was moderately good (ICC (2,k) 0.42, *p* < 0.001), but with significant systematic error (bias of 10.9 mm^3^), even after adopting rigorous measurement criteria. None of the observed CBs was enlarged.

### 3.3. Receiver Operating Characteristics

**Figure 3:**
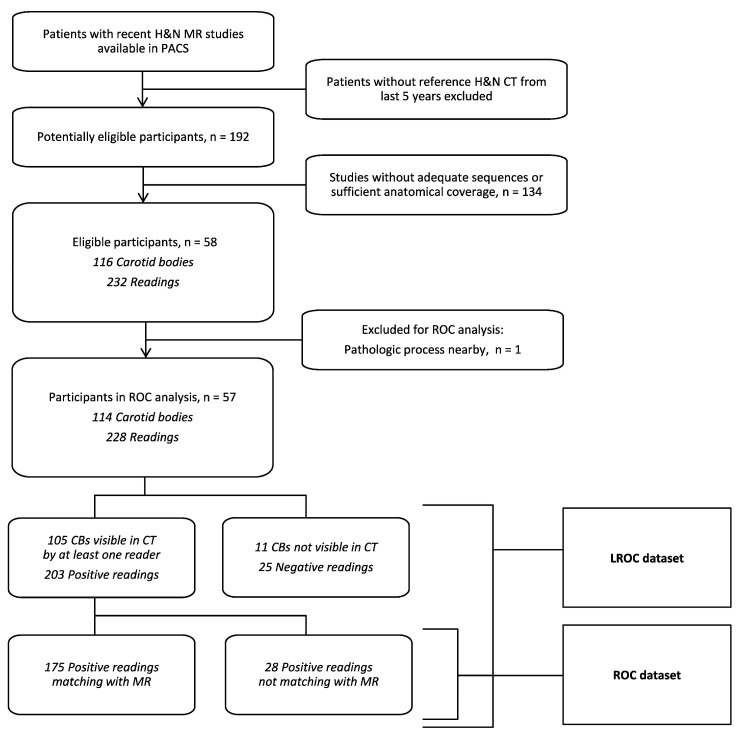
Flow chart. Flow chart depicting patient selection process for the ROC/LROC analysis.

**Figure 4:**
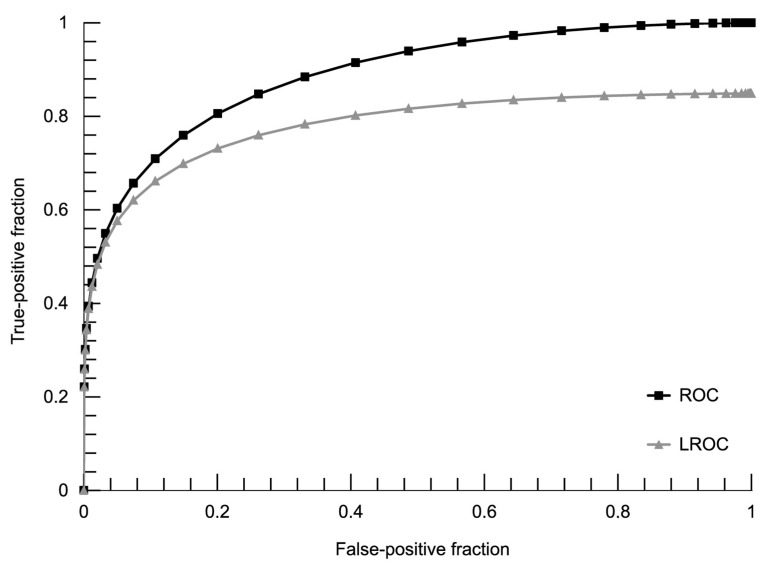
ROC and LROC curves. Calculated ROC and LROC curves were plotted from fit-distribution parameters.

There were 228 readings eligible for analysis (Figure 3 and Table 3). Excluding 25 cases where the CB could not be found on CT images, 203 entries were used to generate an analogous ROC curve with the maximum likelihood of fit a binormal model (Figure 4). Using the ROC cut-off value of two points in our semi-quantitative confidence scale (meaning all discernible structures counted as positives), we achieved specificity of the MR method as 64.3% with 100% sensitivity. The diagnostic performance of the MR method added up to 88.4% of the area under the ROC curve and 78.0% AUC in the LROC algorithm. Details of ROC and LROC processing can be viewed in Supplementary Materials File S2.

## 4. Discussion

In this study, we demonstrated for the first time that CBs could be visualized and assessed with an appropriately designed MR study protocol. Although MR revealed fewer CBs than CT, the diagnostic accuracy was satisfactory, even in LROC analysis. CBs assessed in MR had similar dimensions as described in published anatomical studies [1]. CB morphology in MR approximated the CT examinations. The CB dimensions and volume were significantly larger in MR, probably due to lower spatial resolution and avid enhancement of CBs, causing a partial volume artefact. If the differences in the volume of CBs between CT and MR have a clinical impact should be established in the future. Importantly, there was no significant difference between volumes of CBs of the left and right side in MR, which may be used for comparative assessment.

Instead of CTA, as suggested in some previous papers, we utilized CT examinations in the arteriovenous phase as a reference, more suitable for the assessment of parenchymal structures such as CBs and fully diagnostic [8]. In our study, the arteriovenous phase in CT facilitated visualization of 90% of CBs. In comparison, Jazwiec et al. exposed in CTA only up to 62% of CBs [8], and Nguyen et al. up to 86% of CBs [7]. Cramer et al. managed to detect 91% of expected CBs in CTA [16], comparable with our result. The above data support the usefulness of the arteriovenous phase in CT in the detection of CBs.

There is some bias related to the expected location and number of CBs. We did not observe any developmental anomalies described in the literature, such as duplication or atypical shape [17]. A relatively small study group and a very low incidence of such anomalies probably did not allow us to note these.

The study has some limitations. The first and most important is the lack of anatomical confirmation. However, a study with histopathological verification would be difficult to design from an ethical point of view. Thus, we used the most widely available, non-invasive reference method of visualization, i.e., CT [7,8,16]. Secondly, the study group is small in the context of anatomical investigation. However, the main aim of this study was merely to demonstrate the feasibility of CB visualization with MR. Thirdly, the spatial resolution in MR studies is lower than in CT; however, the size of the applied acquisitional voxel of the VIBE sequence complies with the Nyquist theorem. A higher spatial resolution could be achieved by a 3T scanner and allow for improved detectability and size assessment of CBs. Additionally, some factors might have impacted CT examinations used as the referral method. CT images were calculated with different kernel algorithms. According to previous studies on lung nodules, the kernel algorithm has little effect on volume measurement [18,19]. Lastly, taking into the assumption that all the patients have CBs on both sides of the neck, both modalities in our study failed to recognize all the CBs. Nevertheless, the results are comparable with CTA [16].

We see the need for further studies, not only similar to this one but also to evaluate correlations between MR and other modalities. The MR imaging of CB enlargement caused by various conditions (DM, HT, and CHF) is an exciting challenge. It needs to be emphasized that MR imaging has unquestionable advantages over CT and CTA: a lack of radiation, high soft-tissue contrast resolution, and fewer adverse reactions to macrocyclic CA. The usefulness of MR in the planning of treatment of CBs’ enlargement and employing MR for imaging other chemoreceptors is also worth assessing. Modern MR scanners can offer a relatively short protocol time, and the Dixon sequence provides excellent fatty tissue saturation.

## 5. Conclusions

CBs can be visualized in contrast-enhanced MR studies with good accuracy and inter-observer agreement. CBs assessed in MR had a similar morphology to that described in anatomical studies.

## Figures and Tables

**Table 1:** The semi-quantitative confidence scale for assessment of the carotid bodies.

Score	ROC Threshold Criteria (Both Lists Are Equivalent)
6	Single structure with four typical features
5	Single structure with three typical features
4	Single structure with two typical features OR two structures, one of them with more typical features
3	Single structure with one typical feature OR two structures, both comparable
2	Single structure with no typical features OR three or more comparable structures
1	None structure visible

The features that were taken into consideration: (1) well-defined oval or flame-like shape, (2) typical dimensions, (3) marked enhancement after contrast agent, (4) proximity to the carotid bifurcation (see also Supplementary Materials File S1).

**Table 2:** Comparison of mean carotid body dimensions (in mm) and volume (in mm^3^) in magnetic resonance and computed tomography, with 95% CI in brackets.

	MR	CT	ICC (2,k)	*p* Level
long transverse dimension (mm)	2.7 (2.6–2.8)	2.6 (2.5–2.7)	0.40 (0.10–0.61)	<0.01
short transverse dimension (mm)	2.3 (2.2–2.4)	2.1 (2.0–2.2)	−0.08 (−0.56–0.26)	0.65
longitudinal dimension (mm)	5.8 (5.6–6.1)	5.6 (5.4–5.9)	0.72 (0.57–0.82)	<0.001
volume (mm^3^)	20.8 (19.0–22.7)	19.4 (17.5–21.4)	0.46 (0.17–0.65)	<0.003

The mean degree of enhancement in carotid arteries equalled 191 HU, with a standard deviation of 42 HU. There was no significant correlation between carotid arteries enhancement and reported CB visibility (Spearman’s ranks, *p* = 0.68).

**Table 3:** Frequency summary table of the carotid bodies eligible for ROC analysis.

Method		CT			Total	Confidence Score
		Invisible	Visible			
			Not Matching	Matching		
MR	Invisible	10 (4.39%)	18 (7.89%)	-	28 (12.28%)	1
	Visible	3 (1.32%)	0 (0.0%)	2 (0.88%)	5 (2.19%)	2
		0 (0.0%)	0 (0.0%)	11 (4.82%)	11 (4.82%)	3
		6 (2.63%)	6 (2.63%)	25 (10.96%)	37 (16.23%)	4
		4 (1.75%)	2 (0.88%)	56 (24.56%)	62 (27.19%)	5
		2 (0.88%)	2 (0.88%)	81 (35.53%)	85 (37.28%)	6
Total		25 (10.96%)	28 (12.28%)	175 (76.75%)	228 (100%)	

## Data Availability

Not applicable.

## Supplementary Materials

The following supporting information can be downloaded at: https://www.mdpi.com/article/10.3390/diagnostics13050993/s1, File S1: Detailed assessment protocol of carotid bodies in CT and MRI; File S2: Details of ROC and LROC processing.
