# Supplementary material for: Assessment of the Carotid Bodies in Magnetic Resonance—A Head-to-Head Comparison with Computed Tomography
Source: Diagnostics (Basel). 2023 Mar 5;13(5):993. doi: 10.3390/diagnostics13050993 (PMC10000419; doi:10.3390/diagnostics13050993)

Computed Tomography	Magnetic Resonance
1. Open the most recent head and neck study from patient folder<br>2. Set layout to multi-planar reconstruction<br>3. Load proper sequence :<br>a. first phase after contrast media application<br>b. soft tissues reconstruction algorithm<br>c. layer width less or equal 1.5 mm<br>4. Set Window W 190 C 90 ("liver")<br>5. Hide previous measurements<br>6. Zoom image as necessary<br>7. Set reconstructed layer width to about 1.0 mm<br>8. Localize carotid body on the right side of the neck<br>a. If in doubt, search for confirmation in other sequences from current study<br>9. Align three orthogonal planes with carotid body axes<br>10. Measure longitudinal dimension „z”<br>11. Measure transverse dimensions<br>a. Long axis mark as „y”<br>b. Short (perpendicular) axis mark as „x”<br>12. Create region of interest (ROI) in CCA lumen 1–2 cm below bifurcation<br>13. Missing data annotate as:<br>a. „n/a” – structure truly imperceptible<br>b. „path” – pathologic process in close proximity<br>c. „out” – bifurcation outside field of view<br>d. other feedback put in dedicated comment section<br>14. Repeat steps from 8 to 13 for the left side of the neck	1. Open the most recent head and neck study from patient folder<br>2. Set layout to multi-planar reconstruction<br>3. Load sequence: t1_vibe_dixon_tra_iso_cm_W<br>4. Hide previous measurements<br>5. Zoom image as necessary<br>6. Set reconstructed layer width to about 1.0i mm<br>7. Localize carotid body on the right side of the neck<br>8. Align three orthogonal planes with carotid body axes<br>9. Measure longitudinal dimension „z”<br>10. Measure transverse dimensions<br>a. Long axis mark as „y”<br>b. Short (perpendicular) axis mark as „x”<br>11. Missing data annotate as:<br>a. „n/a” – structure truly imperceptible<br>b. „path” – pathologic process in close proximity<br>c. „out” – bifurcation outside field of view<br>d. other feedback put in dedicated comment section<br>12. Evaluate probability that marked structure will match carotid body location in CT study. Use six-point scale described below.<br>13. Repeat steps from 7 to 12 for the left side of the neck

- **MR studies must be opened after evaluation of CT images has been finished.**
- **Do not compare results between both modalities (single-blinded experiment)**

Points	ROC threshold criteria (both lists are equivalent)	
**6**	Single structure resembling carotid body	
**5**	Single structure, one atypical feature	
**4**	Single structure, two atypical features	Two structures, one of them with more typical features
**3**	Single structure, three atypical features	Two structures, both comparable
**2**	Single structure bearing little resemblance	Three or more comparable structures
**1**	None structure visible	

Typical features list:

1. clearly separated from adjacent structures, maintaining oval or flame-like shape
2. dimensions in rapport with previous anatomic studies (transverse axis 2-4mm, longitudinal up to 8mm)
3. marked enhancement after CM, but to lesser degree then vessel lumen
4. location adjacent to the carotid artery bifurcation

Specific remarks about measurement technique:

1. Longitudinal dimension can be taken either from sagittal or frontal plane, depending on its skew.
2. Longitudinal dimension connects (bright) apex of carotid body with its (usually delineated more poorly) fundus, where it adheres to the CCA.
3. In CT readings darker capsule has to be taken into account
4. In MR readings capsule is not conspicuous, only bright voxels are included

CT transverse

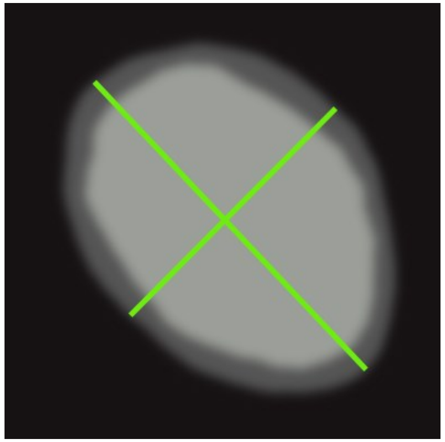

CT longitudinal

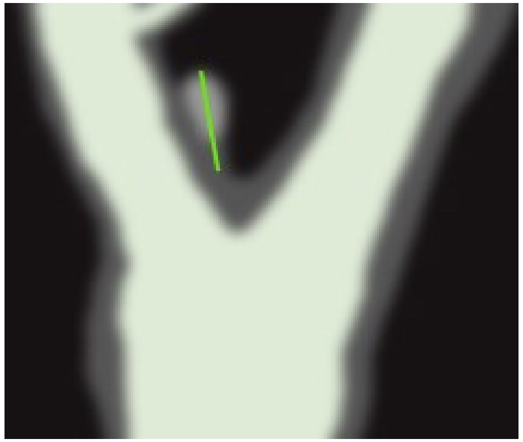

MR longitudinal

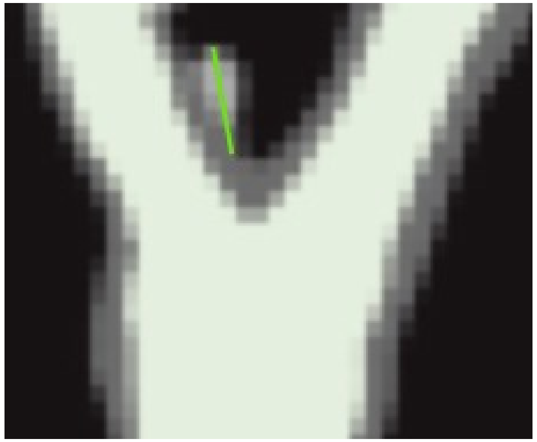

MR transverse

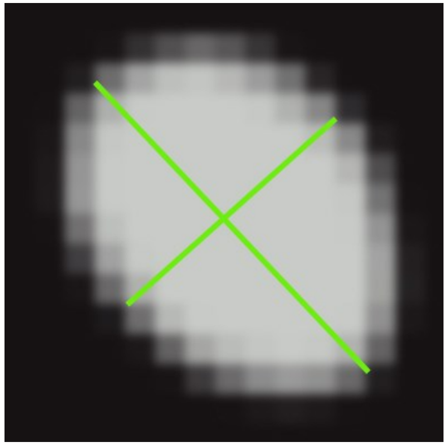

Supplement: Supplementary file 1 [file diagnostics-13-00993-s001.zip › S1_file.pdf]
