# Supplementary material for: Assessment of the Carotid Bodies in Magnetic Resonance—A Head-to-Head Comparison with Computed Tomography
Source: Diagnostics (Basel). 2023 Mar 5;13(5):993. doi: 10.3390/diagnostics13050993 (PMC10000419; doi:10.3390/diagnostics13050993)
Supplement: Supplementary file 1 [file diagnostics-13-00993-s001.zip › S2_file.pdf]

ROC Data Input

Data Format: Frequency Table
Number of Rating Categories: 6

18	0	0	6	2	2
0	2	11	25	56	81

ROC Results

JROCFIT:
Maximum likelihood estimation of a binormal ROC curve from categorical rating data.

DATA CHARACTERISTICS:
Data collected in 6 categories with category 6 representing strongest evidence of positivity (e.g., that abnormality is present).
Number of actually negative cases = 28
Number of actually positive cases = 175

RESPONSE DATA:

Category	1	2	3	4	5	6
Actually negative cases	18	0	0	6	2	2
Actually positive cases	0	2	11	25	56	81

OBSERVED OPERATING POINTS:

FPF:	0.0000	0.0714	0.1429	0.3571	0.3571	0.3571	1.0000
TPF:	0.0000	0.4629	0.7829	0.9257	0.9886	1.0000	1.0000

INITIAL VALUES OF PARAMETERS:
A = 2.7086
B = 1.9125
Z(K): 0.1657 0.2657 0.3657 1.0676 1.4655
LOGL = -262.5308

FINAL VALUES OF PARAMETERS:
Procedure converges after 9 iterations.
A = 3.3401
B = 2.6052
Z(K): 0.3258 0.4488 0.6926 0.9835 1.3198
LOGL = -249.4229

VARIANCE-COVARIANCE MATRIX:

A	0.7417	0.5378	0.1707	0.1646	0.1329	0.0794	0.0123
B	0.5378	0.7882	0.0666	0.0485	-0.0081	-0.0907	-0.1904
Z(1)	0.1707	0.0666	0.0580	0.0542	0.0477	0.0403	0.0318
Z(2)	0.1646	0.0485	0.0542	0.0560	0.0505	0.0449	0.0386
Z(3)	0.1329	-0.0081	0.0477	0.0505	0.0543	0.0542	0.0550
Z(4)	0.0794	-0.0907	0.0403	0.0449	0.0542	0.0653	0.0763
Z(5)	0.0123	-0.1904	0.0318	0.0386	0.0550	0.0763	0.1013

CORRELATION MATRIX:

A	1.0000	0.7034	0.8229	0.8075	0.6625	0.3607	0.0450
B	0.7034	1.0000	0.3114	0.2306	-0.0391	-0.3997	-0.6738
Z(1)	0.8229	0.3114	1.0000	0.9508	0.8502	0.6552	0.4149
Z(2)	0.8075	0.2306	0.9508	1.0000	0.9152	0.7419	0.5126
Z(3)	0.6625	-0.0391	0.8502	0.9152	1.0000	0.9110	0.7418
Z(4)	0.3607	-0.3997	0.6552	0.7419	0.9110	1.0000	0.9375
Z(5)	0.0450	-0.6738	0.4149	0.5126	0.7418	0.9375	1.0000

SUMMARY OF ROC CURVE:
Area = 0.8843
Std. Dev. (Area) = 0.0506

ESTIMATED BINORMAL ROC CURVE WITH ASYMMETRIC 95% CONFIDENCE INTERVAL:

FPF	TPF	95% Conf. Interv.
0.005	0.0004	(0.0000, 0.5539)
0.010	0.0032	(0.0000, 0.6484)
0.020	0.0221	(0.0000, 0.7454)
0.030	0.0593	(0.0000, 0.8011)
0.040	0.1109	(0.0003, 0.8392)
0.050	0.1721	(0.0013, 0.8675)
0.060	0.2385	(0.0041, 0.8895)
0.070	0.3066	(0.0098, 0.9073)
0.080	0.3741	(0.0197, 0.9218)
0.090	0.4390	(0.0349, 0.9339)
0.100	0.5004	(0.0561, 0.9442)
0.110	0.5574	(0.0831, 0.9528)
0.120	0.6098	(0.1159, 0.9602)
0.130	0.6574	(0.1535, 0.9666)
0.140	0.7004	(0.1950, 0.9720)
0.150	0.7389	(0.2392, 0.9766)
0.200	0.8745	(0.4668, 0.9913)
0.250	0.9434	(0.6494, 0.9973)
0.300	0.9759	(0.7715, 0.9993)
0.400	0.9963	(0.8975, 1.0000)
0.500	0.9996	(0.9507, 1.0000)
0.600	1.0000	(0.9760, 1.0000)
0.700	1.0000	(0.9889, 1.0000)
0.800	1.0000	(0.9956, 1.0000)
0.900	1.0000	(0.9989, 1.0000)
0.950	1.0000	(0.9997, 1.0000)

ESTIMATES OF EXPECTED OPERATING POINTS ON FITTED ROC CURVE:

Expected Operating Point (FPF , TPF)	95% C.I. of Lower Bound (FPF , TPF)	95% C.I. of Upper Bound (FPF , TPF)
(0.0935, 0.4609)	(0.0260, 0.0424)	(0.2432, 0.9366)
(0.1627, 0.7817)	(0.0689, 0.2991)	(0.3147, 0.9814)
(0.2443, 0.9377)	(0.1252, 0.6354)	(0.4068, 0.9968)
(0.3268, 0.9850)	(0.1807, 0.8320)	(0.5061, 0.9996)
(0.3723, 0.9936)	(0.2124, 0.8964)	(0.5582, 0.9999)

WARNINGS AND ERROR MESSAGES:
Chi-square goodness of fit not calculated because
some expected cell frequencies are less than 5.
Chi-square goodness of fit not calculated because
some expected cell frequencies are less than 5.

FPF	TPF	Lower	Upper
0.0000	0.0000	0.0000	0.0000
0.0050	0.0004	0.0000	0.5539
0.0100	0.0032	0.0000	0.6484
0.0200	0.0221	0.0000	0.7454
0.0300	0.0593	0.0000	0.8011
0.0400	0.1109	0.0003	0.8392
0.0500	0.1721	0.0013	0.8675
0.0600	0.2385	0.0041	0.8895
0.0700	0.3066	0.0098	0.9073
0.0800	0.3741	0.0197	0.9218
0.0900	0.4390	0.0349	0.9339
0.1000	0.5004	0.0561	0.9442
0.1100	0.5574	0.0831	0.9528
0.1200	0.6098	0.1159	0.9602
0.1300	0.6574	0.1535	0.9666
0.1400	0.7004	0.1950	0.9720
0.1500	0.7389	0.2392	0.9766
0.2000	0.8745	0.4668	0.9913
0.2500	0.9434	0.6494	0.9973

0.3000	0.9759	0.7715	0.9993
0.4000	0.9963	0.8975	1.0000
0.5000	0.9996	0.9507	1.0000
0.6000	1.0000	0.9760	1.0000
0.7000	1.0000	0.9889	1.0000
0.8000	1.0000	0.9956	1.0000
0.9000	1.0000	0.9989	1.0000
0.9500	1.0000	0.9997	1.0000
1.0000	1.0000	1.0000	1.0000

LROC Data Input

OBSERVER:LB&TN
CONDITION:MRvsCT
REPLICATION:
NDIST: 1
NCAT: 6

DISTRIBUTION: 0

1	0	10
2	0	3
3	0	0
4	0	6
5	0	4
6	0	2

DISTRIBUTION: 1

1	18	0
2	0	2
3	0	11
4	6	25
5	2	56
6	2	81

LROC Results

OBSERVER:LB&TN
CONDITION:MRvsCT
REPLICATION:

NDIST: 1 Number of target distributions
NCAT: 6 Number of rating categories
NOLOC: 0 Number of categories without localization

Chance Correct Location (qset) values:
1
0.000

DISTRIBUTION: 0 Non-target distribution

Rating	Frequency
1	10
2	3
3	0
4	6
5	4
6	2

DISTRIBUTION: 1 Target distribution

Rating	Non-hit	Hit
1	18	0
2	0	2
3	0	11
4	6	25
5	2	56
6	2	81

Procedure has estimates of model parameters
Number of interations = 7
Stopping index (tnet) = 0.00080599 lambda = 0.10000000000000E-06
Number of distributions = 2

Mean of distributions

Fit	1.9874	0.0000
Inital	2.4366	0.0000

Sigma of distributions

Fit	1.6483	1.0000
Inital	2.0000	1.0000

```
    Boundaries
Fit      0.1822   0.3284   0.5970   1.2947   2.3549
Inital   0.0463   0.1864   0.2338   0.8957   1.4464

     Distibution parameter values
Target dist #     1
Mean           1.98744  0.00000
    Std err    0.23245  0.00000
Sigma          1.64826  1.00000
    Std err    0.24821  0.00000

Boundaries     0.18216  0.32844  0.59704  1.29473  2.35489
    Std err    0.14203  0.13720  0.13131  0.13987  0.23873

Used numerical integration from parameter values
Target dist #     1
AreaROC        0.8901
   std err     0.0146
AreaLROC       0.7801
Loc Accuracy   0.8487
   std err     0.0237

Direct calculation from parameter values
Dloc            1.4579
   std err      0.1427
Prob Local      0.8487
   std err      0.0237

Moments from distribution parameters of LROC model
Target dist #     0        1
Mean           0.0000   2.1292
St. dev.       1.0060   1.4553
Skew           0.0000   0.2922
Kurtosis       0.1853  -6.5201
Implied d-a    0.0000   1.7054
Implied Az     0.5000   0.8861

Loglikelihood and chi-squared
logd1,logd2    -392.29388    -334.75824
logL1,logL2      64.79968      16.44430
chi1,chi2        61.33489      22.84165

Curves from fit distribution parameters
 False Pos      Tar 1
  1.00000   1.0000 0.8487
  0.99859   1.0000 0.8487
  0.99733   1.0000 0.8487
  0.99512   1.0000 0.8487
  0.99140   1.0000 0.8487
  0.98542   0.9999 0.8487
  0.97614   0.9998 0.8486
  0.96234   0.9996 0.8485
  0.94262   0.9991 0.8483
  0.91556   0.9983 0.8479
  0.87990   0.9967 0.8471
  0.83476   0.9940 0.8458
  0.77989   0.9896 0.8436
  0.71582   0.9829 0.8402
  0.64398   0.9728 0.8349
  0.56662   0.9586 0.8271
  0.48662   0.9395 0.8163
  0.40714   0.9147 0.8018
  0.33133   0.8841 0.7830
  0.26187   0.8476 0.7596
  0.20076   0.8058 0.7314
  0.14911   0.7594 0.6986
```

0.10719	0.7094	0.6614
0.07452	0.6570	0.6205
0.05006	0.6034	0.5766
0.03247	0.5494	0.5306
0.02033	0.4960	0.4833
0.01228	0.4440	0.4358
0.00715	0.3939	0.3888
0.00401	0.3463	0.3432
0.00217	0.3014	0.2997
0.00113	0.2597	0.2587
0.00057	0.2214	0.2209
0.00000	0.0000	0.0000

Standard Error -- Correlation matrix

Mean and sigma part of matrix

	m2	s2
m2	0.23245	0.62965
s2	0.62965	0.24821

Boundary part of matrix

	b1	b2	b3	b4	b5
b1	0.14203	0.90634	0.75935	0.42564	0.05911
b2	0.90634	0.13720	0.83954	0.49071	0.11443
b3	0.75935	0.83954	0.13131	0.62594	0.23905
b4	0.42564	0.49071	0.62594	0.13987	0.64027
b5	0.05911	0.11443	0.23905	0.64027	0.23873

Mean and sigma by boundary

	m2	s2
b1	0.13288	-.30032
b2	0.18338	-.26014
b3	0.29275	-.15701
b4	0.61223	0.25065
b5	0.81157	0.71692
